# Goiter in a Patient with Pulmonary Arterial Hypertension Treated with Epoprostenol

**DOI:** 10.1155/2020/1617253

**Published:** 2020-01-23

**Authors:** Shaadi Abughazaleh, Zeenat Safdar

**Affiliations:** Department of Pulmonary-Critical Care Medicine, Houston Methodist Hospital, Weill Cornell College of Medicine, Houston, TX, USA

## Abstract

A 35-year-old female with pulmonary arterial hypertension (PAH) who presented with complaints of progressively worsening dysphagia, facial swelling, and shortness of breath, was found to have a large goiter. In patients treated with epoprostenol for long periods of time, thyroid disease is common. Most cases of thyroid disease describe thyrotoxicosis and hyperthyroid statues, but our case was a patient on long term IV epoprostenol presenting with a superior vena cava-syndrome (SVC) like appearance and airway compromise found to have a goiter incidentally during workup.

## 1. Introduction

Pulmonary arterial hypertension is a rare disease which occurs in 5–50 per one million adults [1–3]. This life-threatening disease occurs 2–4x more commonly in females compared to men [3, 4]. Although with new treatment modalities PAH-related hospitalizations and death are decreasing during the last decade [5, 6], many of PAH medications have significant side effects. A number of studies have documented that in patients diagnosed with hyperthyroidism, 35–47% were also found to have PAH. Whereas, studies have shown that anywhere between 22.5% and 49% of PAH patients have hyperthyroidism [7], although a clear causality has not yet been described. We present a case of PAH patients on long-term intravenous epoprostenol infusion who presented with an enlarging goiter with compression symptoms.

## 2. Case

A 35-year-old African-American female was diagnosed with PAH in 2010, five-months after the birth of her 3^rd^ child. She started noticing progressive shortness of breath including an episode of syncope while walking into a shower. She denied any drug abuse or anorexigen use. She saw a number of doctors including neurologist, cardiologist and pulmonologist and remembers receiving “every test you can think of” before she was referred to pulmonary hypertension specialist. Her echocardiogram (ECHO) showed elevated right ventricular pressure (Table 1). She underwent full pulmonary hypertension work up including a right heart catheterization (RHC) that demonstrated a mean pulmonary artery pressure of 52 mmHg (Table 2). Computed tomography of chest showed an enlarged pulmonary trunk measuring 3.7 cm and borderline cardiomegaly but no interstitial changes. The ventilation-perfusion scan showed a low probability of pulmonary embolism. She was started on sildenafil and ambrisentan and inhaled treprostinil was subsequently added with only marginal improvement of symptoms. Therefore, she was rapidly transitioned to intravenous epoprostenol. With this, she noticed a marked improvement in her shortness of breath and functionality. In 2015, she was evaluated by ophthalmology for headaches due to concern for nonarteritic anterior ischemic optic neuropathy (NAION), and sildenafil was discontinued. In 2018, she underwent a repeat RHC for worsening shortness of breath that showed mean PAP of 60 mmHg, right atrial pressure of 5 mmHg, cardiac output of 2.78 liters per minute and MVO2 of 59%. She started complaining of shortness of breath, neck swelling and pain, dyspnea, dysphagia and occasional dysphonia. Physical exam showed evidence of an enlarging neck mass resulting in dyspnea and anxiety. There was progressive worsening in her shortness of breath, which was not attributable to PAH, so endocrinology was consulted. Thyroid studies showed T4 = 1.4 ng/dL (0.9–1.7 ng/dL), TSH = 0.34 *µ*IU/mL (0.27–4.20 *µ*IU/mL), undetectable thyroglobulin antibody, elevated thyroglobulin serum at 147.6 ng/mL (1.3–31.8 ng/mL) and thyroperoxidase antibody at 70.4 IU/mL (0.0–9.0 IU/mL) (seen in Table 3). Further lab workup showed stable electrolytes and baseline renal function.

**Figure 1:**
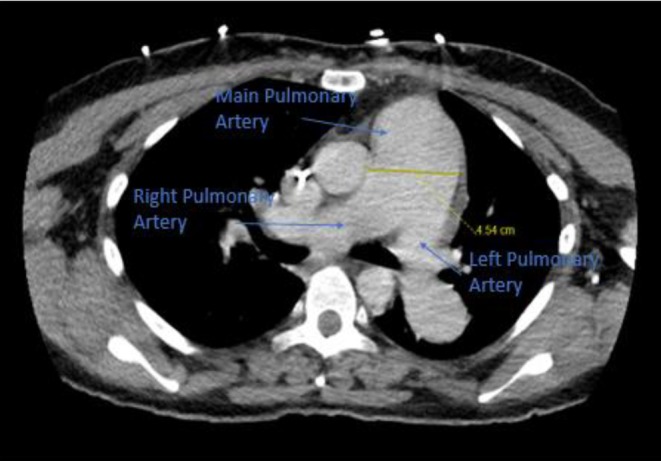
CT chest with contrast showing enlarged pulmonary artery measuring 4.5 cm.

**Figure 2:**
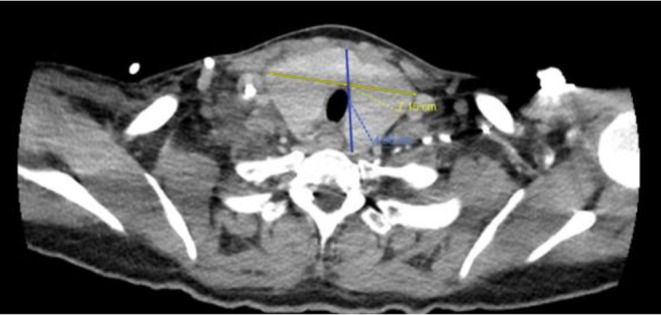
CT chest with contrast showing enlarged thyroid gland approximately 7 × 5 cm in size.

CT chest (Figure 1) and neck (Figure 2) were done for initial evaluation for her in the setting of dyspnea, neck swelling and pain which showed thyromegaly with 1.6 cm isthmus nodule and 2 cm left thyroid (seen in Figure 2). 

Subsequent fine needle aspiration of both nodules showed no evidence of malignancy and only findings of benign follicular consistent with an adenomatous nodule. Radioactive iodine uptake test showed absence of thyroid uptake, thyroid stimulating immunoglobulin was negative, and there was no evidence of Graves' disease or hyper-functioning thyroid. In the setting of laboratory euthyroidism and a goiter with the characteristics mentioned above, she was diagnosed with silent (painless) thyroiditis. With this diagnosis, radioactive iodine ablation was not deemed necessary as she was in the euthyroid state. However, patient continued to have progressively worsening compressive symptoms from her enlarging goiter. After discussion with Otolaryngology and Endocrinology, it was recommended that she have her goiter removed.

**Figure 3:**
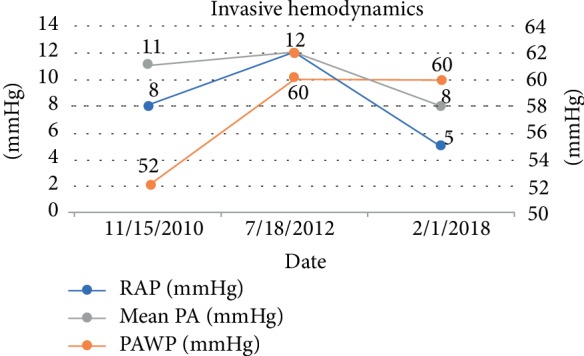
Figure representing changes in RAP, Mean PAP, and PAWP at time points leading up to her procedure.

**Figure 4:**
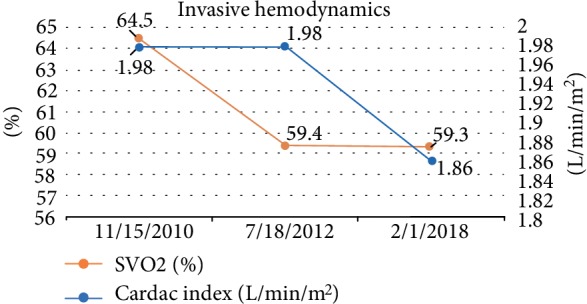
Figure representing changes in SVO2 and cardiac index at time points leading up to her procedure.

She was admitted for thyroidectomy and her PAH meds were optimized [riociguat was added and rapidly up-titrated to 2.5 mg TID; epoprostenol dose at 48 ng/kg/min and ambrisentan 10 mg daily were continued]. Multidisciplinary meetings were held with cardiovascular anesthesia, ICU team and surgeons regarding the importance of continuing epoprostenol during this procedure along with recommendations to use inhaled agents, avoiding propofol during induction. She successfully underwent thyroidectomy without any complications and with full post-operative recovery. Following her surgery, ECHO showed estimated RVSP of 65–70 mmHg, severely enlarged right ventricle with severely depressed systolic function and right atrial pressure of 5 mmHg. Patient was continued on epoprostenol 48 ng/kg/min, ambrisentan 10 mg daily and riociguat 2.5 mg three times per day with outpatient follow-up. At 6 month follow-up, patient self-reports WHO Class III functional status. Patient's RAP, Mean PAP, and PAWP over time are shown in Figure 3. Patient's SVO2 and Cardiac Index over time are shown in Figure 4. She continues to be on stable doses of epoprostenol and riociguat with plans for future RHC and gradual increase in epoprostenol as tolerated.

## 3. Discussion

This patient was treated with intravenous epoprostenol who presented with an enlarging goiter. Seronegative thyrotoxicosis, diffuse goiters, and homogenous uptake on thyroid scintigraphy in patients on long term epoprostenol therapy has been reported by Chadha et al. [8]. It was found that 6.7% of patients with PAH treated with PGI2 had thyroid-stimulating immunoglobulin-negative thyrotoxicosis in the absence of other factors that could be contributing to their hyperthyroid state. This number was significantly greater than expected rates in the general population [8].

It is important to distinguish patients treated with IV Epoprostenol from patients who present with PAH and thyroid disease, two diseases that have known associations. It has been proposed that the direct influence of thyroid hormone on the pulmonary vasculature can cause increased pressures. In multiple case reports, patients with Graves' disease saw decreases in, and at times, normalization of PASP after the treatment of thyroid disease [7]. In a study comparing epoprostenol and endothelin receptor antagonists in the development of thyrotoxicosis in patients with PAH, it was found that when compared to patients on combination therapy, patients being treated with *only* epoprostenol had significantly higher odds of thyrotoxicosis [9]. This leads practitioners to believe that thyrotoxicosis may be promoted by epoprostenol and inhibited by endothelin receptor antagonists which are often used in the treatment of PAH.

In this case, the patient had been treated with IV epoprostenol for more than 6 years and was found to have a goiter causing airway compression and an SVC-syndrome-like manifestation with facial swelling, shortness of breath and difficulty swallowing foods. Prior thyroid studies and imaging completed prior to initiation of epoprostenol were unremarkable. Upon further investigation, patient was found to be in a euthyroid state which was unique when compared to the hyperthyroid state many of the previous case reports had described. This case's purpose is to make practitioners who treat PAH with epoprostenol aware of not only the association with thyrotoxicosis and hyperthyroid states but also aware that goiters presenting with an SVC-syndrome like appearance and airway compression may occur with long-term use of epoprostenol.

## Figures and Tables

**Table 1:** Non-invasive hemodynamics.

Date	RAP (mmHg)	RVSP (mmHg)	CI (L/min/m^2^)	HR (bpm)	Mean BP (mmHg)
11/2/10	15	87	1.29	52	104
2/16/11	15	80	1.61	73	110.5
3/6/12	10	105	1.36	91	90
4/8/13 (pre-flolan)	10	100	2.4	78	86
7/23/13 (post-flolan)	15	133	2.1	95	88
6/6/14	5	81	2.1	89	82.5
3/4/15	5	110	2.35	—	—
10/6/15	10	91	1.7	—	—
8/2/18 (pre-surgery)	5	75	2.0	84	66.5
8/5/18 (post-surgery)	5	70	Not reported	80	74.5
11/14/18	10	100	3.6	91	81.5
2/20/19	5	90	4.1	92	72

**Table 2:** Invasive hemodynamics.

Date	RAP (mmHg)	Mean PA (mmHg)	PAWP (mmHg)	Cardiac index (L/min/m^2^)	SVO2 (%)
11/15/10	8	52	11	1.98	64.5
7/18/12	8–12	60	8–12	1.98	59.4
2/1/18 (Pre-surgery)	5	60	8	1.86	59.3

**Table 3:** Thyroid studies.

T4	1.4 *µ*g/dL (4.5–11.7 *µ*g/dL)
Free T4	1.0 mg/dL (0.9–1.7 ng/dL)
TSH	0.34 *µ*IU/mL (0.27–4.20 *µ*IU/mL)
Thyroglobulin antibody	Undetectable
Thyroglobulin serum	147.6 ng/mL (1.3–31.8 ng/mL)
Thyroperoxidase antibody	70.4 IU/mL (0.0–9.0 IU/mL)
